# Effects of cognitive behavioral and psychological intervention on social adaptation, psychological resilience and level of hope in patients with nasopharyngeal carcinoma in radiotherapy

**DOI:** 10.12669/pjms.40.1.7421

**Published:** 2024

**Authors:** Xiaohui Liu, Ce Wang, Yanhong Li, Yue Wang

**Affiliations:** 1 Xiaohui Liu, Department of Radiotherapy, Affiliated Hospital of Hebei University, Baoding 071000, Hebei China; 2 Ce Wang, Department of Radiotherapy, Affiliated Hospital of Hebei University, Baoding 071000, Hebei China; 3 Yanhong Li, Department of Radiotherapy, Affiliated Hospital of Hebei University, Baoding 071000, Hebei China; 4 Yue Wang, Department of Radiotherapy, Affiliated Hospital of Hebei University, Baoding 071000, Hebei China

**Keywords:** Cognitive behavioral and psychological intervention, nasopharyngeal carcinoma, Social adaptation, Psychological resilience, Level of hope Treatment

## Abstract

**Objective:**

To evaluate the effects of cognitive behavioral and psychological intervention(CBPI) on social adaptation, psychological resilience, and the level of hope in patients with nasopharyngeal carcinoma(NPC) in radiotherapy.

**Methods:**

This is application research. Eighty patients undergoing radiotherapy for NPC at Affiliated Hospital of Hebei University from November 20, 2020 to November 15, 2022 were randomized into control and study groups at a 1:1 ratio. While the control group was provided with standard specialized nursing care, the study group was offered CBPI in addition to the exact nursing care. Differences in quality of life, psychological resilience, level of hope, emotional state, and patient satisfaction between the groups were compared and analyzed before and after treatment.

**Results:**

After an intervention, significantly improved physical, mental, and social functions and material living conditions were observed in the study group compared with the control group (all *p*< 0.05). Although both groups scored higher on the selected psychological resilience scale following the intervention, the study group showed better results as compared to control group in adaptability, tenacity, control, and goal orientation (all *p*< 0.05). While both groups had elevated scores of temporality and future, interconnectedness, and positive readiness and expectancy at the end of the intervention, the improvements were more pronounced in the study group (all *p*< 0.05).

**Conclusion:**

CBPI supports radiotherapy for NPC by improving patients’ quality of life, confidence in treatment, the hope of recovery, psychological resilience, anxiety, depression, and patient satisfaction. Therefore, this treatment strategy is worthy of wide application in clinical settings.

## INTRODUCTION

Nasopharyngeal carcinoma(NPC) is a clinically common malignancy of the head and neck^1^ closely related to the EB virus (EBV).^2^ Most patients present with highly malignant NPC that progresses rapidly and metastasizes easily to distant sites.^3^ With the aid of contemporary and emerging specialized imaging technologies and leading-edge radiotherapy techniques, radiation has become the mainstay of NPC treatment.^4^ However, radiotherapy is reported to profoundly affect the quality of life (QoL) as it causes discomfort or pain and increases anxiety and depression levels.^5^ Ahmad et al.^6^ Pointed out that cognitive biases, negative emotions, and low resilience contributing to the reduced therapeutic alliance and long-term QoL are generally observed in patients undergoing cancer treatment. In the past, standard specialized nursing care was essentially functional nursing that focuses on physical support, in which context mental health nursing was absent or delivered verbally without specific purpose as patients’ psychological needs were overlooked. Fundamental changes have taken place recently with the continuous development of the biopsychosocial model. The importance of cognitive behavioral and psychological intervention (CBPI) has been gaining clinical recognition as a decisive part of holistic nursing. CBPI is defined as an active nursing intervention using psychological techniques and theories to maintain or improve a patient’s mental health throughout treatment.^7^ Therefore, CBPI was applied to NPC treatment and produced favorable outcomes.

## METHODS

This is application research involving eighty patients who received radiotherapy at Affiliated Hospital of Hebei University due to nasopharyngeal carcinoma (NPC) from November 20, 2020 to November 15, 2022. These patients were randomly divided into two groups (n= 40 each). The study group consisted of 23 male and 17 female patients aged between 47-73 years (mean age, 60.35 ± 13.42 years), while the control group had 21 males and 19 females’ patients at the age of 50 to 72 (mean age, 61.27 ± 11.36 years). There was no significant difference in demographic characteristics, suggesting high comparability between the two groups (Table-I).

**Table-I:** Demographic characteristics of the study group versus the control group (*χ̅*±*S*, n = 40).

Indicator	Study group	Control group	t/χ^2^	p
Age, yrs	60.35±13.42	61.27±11.36	0.33	0.74
Male, n (%)	23(57.5%)	21(52.5%)	0.20	0.65
Pathological type				
Poorly differentiated squamous-cell carcinoma, n (%)	8(20%)	6(15%)	0.35	0.56
Moderately differentiated squamous-cell carcinoma, n (%)	17(42.5%)	21(52.5%)	0.80	0.37
Well-differentiated squamous cell carcinoma, n (%)	15(37.5%)	13(32.5%)	0.22	0.64
Highest education level				
Elementary school/none, n (%)	6(15%)	5(12.5%)	0.11	0.75
Middle/high school, n (%)	18(45%)	21(52.5%)	0.45	0.50
Tertiary education, n (%)	16(40%)	14(35%)	0.21	0.64
Disease course, yrs	2.03±0.82	1.96±0.70	0.41	0.68

p> 0.05.

### Ethical Approval

The study was approved by the Institutional Ethics Committee of Affiliated Hospital of Hebei University (No.: HDFYLL-KY-2022-006; date: October 15, 2022), and written informed consent was obtained from all participants.

### Inclusion criteria:

- Meeting the diagnostic criteria for NPC;^8^
- Definite pathological diagnosis by biopsy or surgery;
- Under 75 years of age;
- Normal cognition and communication ability, and active cooperation on treatment and nursing plans;
- Estimated survival > 12 months;
- Conscious and mental disorder-free;
- Complete clinical data.

### Exclusion criteria:

- Severe comorbidities affecting the heart, liver, lung, kidney, and brain; coexisting malignancies in other sites;
- Preexisting mental disorders or concurrent communication disorders;
- Estimated survival< 12 months;
- Inadequate cooperation with investigators; and early withdrawal from the treatment program.
- Malignant tumors in other sites of the body at the same time.

### Methods

The control group was given standard specialized nursing care: after admission, the patient was comprehensively informed of any warnings, precautions, or measures to be taken regarding radiotherapy for NPC; the treatment program was explained in detail upon request of the patient and his/her family; related questions raised by the patient and his/her family were answered sensibly to facilitate the development of a proper understanding of NPC radiotherapy, dispel their fears and nervousness about the disease, and create a trustworthy image for the good therapeutic alliance; guidelines for diet and sleep were developed to ensure a healthy routine and a good sleep environment; oral and nasal care, and specialized nursing intervention for radiated skin and difficulty in the opening mouth were provided.

In addition to the said nursing plan, the study group was provided with CBPI composed of cognitive, behavioral, and emotional intervention activities. The cognitive intervention was designed for patients who had a fundamental misunderstanding of NPC radiotherapy to remove their worries about the disease and radiotherapy by illuminating the pathogenesis of NPC and highlighting the significance of radiotherapy in NPC treatment. Medical practitioners were assigned to show patients around the radiotherapy center before treatment to enhance therapeutic alliance. The behavioral intervention was defined as useful strategies leading patients to a healthier lifestyle. Adequate exercise, such as strolling, jogging, and other activities, was recommended to enhance physical fitness for radiotherapy. Patients were encouraged to take up enjoyable hobbies, which help avoid excessive concern over their conditions.

A balanced diet was designed to ensure adequate nutrition intake. Clear instructions were given to help patients learn how to relax muscles and breathe deeply. The emotional intervention was implemented in various forms. Strong emotional support was enlisted via effective communication with every patient’s family member(s). Psychological counseling was given to patients to understand their emotional needs, encourage the direct expression of negative emotions, and provide necessary interpretation and guidance according to individual trait emotions. Patient mutual support groups were set up to facilitate communication between patients, further bolster confidence in radiotherapy, and improve compliance with prescribed medication by those who were administered analgesics due to intense pain during radiotherapy.

### Outcome measures

The two groups were evaluated on various aspects before and three months after intervention. Pre- and post-intervention QoL was measured using the Generic Quality of Life Inventory-74 (GQOLI-74)^9^, which rates physical, mental, and social functions, and material living conditions based on 20 questions, specifically 74 items on a 5-point scale (1-5), with a higher score denoting greater improvement. Pre-and post-intervention psychological resilience was assessed using the Connor-Davidson Resilience Scale (CD-RISC)^10^ containing 25 items concerning adaptability, tenacity, control, and goal orientation, each rated on a five point scale ranging from zero (not true at all) to four (true nearly all of the time). The total score can be 0-100, with higher scores indicative of higher levels of resilience. Pre and post-intervention levels of hope were measured with the Herth Hope index (HHI)^11^, a 12-item scale covering three dimensions, including temporality and future, interconnectedness, and positive readiness and expectancy. During the six-months follow-up of this study, the survival rate was 100%.

The global HHI score can be 12 to 48, with single-item scores ranging from one to four, and a higher score indicating a higher level of hope. Pre- and post-intervention emotional state was tested using the Self-Rating Anxiety Scale (SAS) and the Self-Rating Depression Scale (SDS)^12^, and lower scores suggest a better emotional state. Pre and post-intervention patient satisfaction was investigated using the short-form Patient Satisfaction Questionnaire (PSQ-18).^13^ Levels of satisfaction were classified as “very satisfied”, “more than satisfied”, “satisfied”, “uncertain”, and “dissatisfied”. The overall percentage of patients satisfied with the treatment program was calculated by dividing the number of patients responding “very satisfied”, “more than satisfied”, and “satisfied” by the total number of patients surveyed and multiplying the result by 100.

### Statistical analysis

All data were processed and analyzed using SPSS 20.0, where measurement data were expressed by (*χ̅*±*S*). Intergroup comparisons were examined by independent-samples t-test, while intragroup comparisons were evaluated by paired t-test. Comparisons of percentages were performed by chi-square test. A value of *p*< 0.05 was considered significant.

## RESULTS

Before the intervention, the two groups lacked significant differences in physical, mental, and social functions, as well as material living conditions (all *p*> 0.05). After the intervention, the study group showed significantly greater improvement in the said dimensions of QoL (all *p*< 0.05) (Table-II).

**Table-II:** Intergroup comparison of pre-and-post-intervention QoL (*χ̅*±*S*, n = 40).

Indicator		Study group	Control group	t	p
Physical function	Pre-intervention	43.72±7.54	43.61±7.48	0.07	0.95
	Post-intervention*	52.86±8.21	48.37±7.63	2.53	0.01
Mental function	Pre-intervention	42.47±7.46	43.02±7.83	0.32	0.75
	Post-intervention*	50.71±7.26	46.84±7.95	2.27	0.03
Social function	Pre-intervention	54.46±6.57	55.02±6.31	0.39	0.70
	Post-intervention*	62.58±8.04	58.66±8.43	2.13	0.03
Material living conditions	Pre-intervention	45.47±8.36	45.19±8.03	0.22	0.84
	Post-intervention*	55.25±8.06	51.04±8.33	2.30	0.02

* p < 0.05.

Before the intervention, no significant difference existed between the two groups regarding adaptability, tenacity, control, or goal orientation (all *p*> 0.05). After the intervention, the study group displayed significantly more pronounced improvement in the above-mentioned indicators of psychological resilience (all *p*< 0.05) (Table-III).

**Table-III:** Intergroup comparison of pre-and-post-intervention psychological resilience (*χ̅*±*S*, n = 40).

Indicator		Study group	Control group	t	p
Adaptability	Pre-intervention	15.62±3.41	15.70±3.53	0.10	0.92
	Post-intervention*	22.17±4.76	18.64±4.09	3.56	0.00
Tenacity	Pre-intervention	13.18±2.03	13.20±2.33	0.04	0.97
	Post-intervention*	21.08±4.16	17.05±3.29	4.81	0.00
Control	Pre-intervention	12.40±3.15	13.04±3.06	0.92	0.36
	Post-intervention*	23.27±4.82	17.33±4.35	5.80	0.00
Goal orientation	Pre-intervention	13.74±2.11	13.59±2.63	0.28	0.78
	Post-intervention*	21.86±3.36	15.81±3.47	7.92	0.00

* p < 0.05.

Before the intervention, there was no significant difference between the two groups as to scores for temporality and future, interconnectedness, and positive readiness and expectancy (all *p*> 0.05). After the intervention, the study group scored significantly higher on the said subscales than the control group (all *p*< 0.05) (Table-IV).

**Table-IV:** Intergroup comparison of pre-and-post-intervention levels of hope (*χ̅*±*S*, n = 40).

Indicator		Study group	Control group	t	p
Temporality and future	Pre-intervention	16.27±3.24	16.36±3.18	0.21	0.83
	Post-intervention*	33.84±8.55	25.43±8.62	3.43	0.00
Interconnectedness	Pre-intervention	17.38±3.74	17.73±4.29	0.39	0.70
	Post-intervention*	35.16±8.40	26.31±8.91	4.57	0.00
Positive readiness and expectancy	Pre-intervention	18.47±4.03	18.19±4.01	0.32	0.76
	Post-intervention*	42.75±10.11	37.46±9.41	3.35	0.00

* p < 0.05.

The two groups did not differ greatly in the pre-intervention SAS and SDS scores (*p* > 0.05, respectively). At the end of the intervention, the SAS and SDS scores were significantly reduced in the study group compared with the control group (*p* < 0.05, respectively) (Table-V). Patient satisfaction was 95% in the study group and 77.5% in the control group, suggesting a significant difference between the two groups (*p* < 0.05) (Table-VI).

**Table-V:** Intergroup comparison of pre-and-post-intervention mental state (*χ̅*±*S*, n = 40).

Indicator		Study group*	Control group	t	p
SAS	Pre-intervention	53.32±7.34	52.89±6.97	0.27	0.80
	Post-intervention*	43.37±6.28	47.85±6.04	3.25	0.00
SDS	Pre-intervention	54.63±6.07	55.02±6.74	0.28	0.78
	Post-intervention*	41.68±6.26	45.87±6.12	3.03	0.00

* p < 0.05.

**Table-VI:** Intergroup comparison of patient satisfaction (*χ̅*±*S*, n =40).

Group	Very satisfied	More than satisfied	Satisfied	Uncertain	Dissatisfied	Overall patient satisfaction*
Study group	28	7	3	2	0	38(95%)
Control group	20	6	5	3	6	31(77.5%)
*c^2^*						4.11
*p*						0.02

* p < 0.05.

## DISCUSSION

The present study has validated the utility of cognitive behavioral and psychological intervention (CBPI) for patients undergoing radiotherapy for nasopharyngeal carcinoma (NPC) to improve social adaptation by revealing the significant differences between the study and control groups in scores for post-intervention physical, mental, and social functions, material living conditions, SAS, and SDS (all *p*< 0.05). A possible explanation for these results is that CBPI had improved patients’ knowledge of NPC as well as their ability to cope with negative emotions by negative emotion detection and health education before treatment, leading to better adaptability to radiotherapy and response to related psychological stress.

Potential adverse reactions caused by radiotherapy are associated with undermined confidence in the treatment and reduced psychological resilience. Higher levels of psychological resilience indicate that patients can cope mentally or emotionally with stressful or traumatic experiences and rebuild confidence in rehabilitation.^14^ Psychological resilience is regarded as a positive psychological trait that keeps an individual rapidly adapting to and bouncing back from adversities. In this study, post-intervention psychological resilience scores regarding adaptability, tenacity, control, and goal orientation were significantly elevated in the study group compared with the control group (all *p*< 0.05).

This study demonstrates the positive effects of CBPI on the psychological resilience of patients receiving radiotherapy for NPC. In this regard, CBPI potentially broadens patients’ understanding of their conditions through negative emotion monitoring and health education and motivates family members to keep company with patients, which helps allay negative emotions and boost confidence in treatment.^15^ In addition, progressive relaxation training was designed to divert patients’ attention, aid relaxation, dispel negative emotions, and instill confidence and courage into their minds to overcome adversities.

Hope denotes a firm belief in life, which reflects positively on one’s behavior and disposition. Patients with high levels of hope are shown to have greater confidence in treatment.^16^ The study group was treated with CBPI and achieved significantly better scores for temporality and future, interconnectedness, and positive readiness and expectancy compared with the control group (all *p*< 0.05). These results can be ascribed to the patient mutual support group model within the CBPI framework, where volunteers share their treatment plans and related information with other patients, allowing them to banish the feeling of uncertainty, build up confidence in treatment, and become hopeful about a good prognosis.^17^ In terms of patient satisfaction, the significant difference between the study and control groups (95% vs. 77.5%, *p*< 0.05) suggests that CBPI can substantially raise the level of satisfaction among patients undergoing radiotherapy for NPC, and the underlying mechanism seems to be linked with higher levels of confidence and hope and improved physical and mental health following CBPI.

NPC, as a frequently occurring malignant tumor, has a pathogenesis closely associated with environmental, hereditary, and lifestyle factors, and the number of patients affected by NPC is increasing every year with changing lifestyles and living conditions.^18^ Early-stage NPC has no distinct clinical presentations but common symptoms such as blood in nasal mucus, sinus congestion, ear stuffiness, and headaches. The disease hence spreads insidiously to other organs, which may result in a grave threat to patients’ life or health.^19^ Although radical radiotherapy represents an important treatment for NPC 20, inappropriate publicity of cancer and radiotherapy has aroused anxious and depressive emotions for the approach in most patients with NPC. Meanwhile, patients lack confidence in radiotherapy in that limited by current medical technologies, it potentially exacerbates fatigue, nausea, and vomiting and reduces QoL during the treatment course. It is therefore a priority of the nursing plan to ease anxiety and depression and improve QoL during radiotherapy.^21^ Schrepf et al^22^ believed that affective and cognitive disorders in patients with cancer are attributable to diagnosis-related painful experiences, impaired QoL, and side effects of primary care-which exert a huge impact on one’s mental health. Cancer patients are shown to fear a relapse.^23^

Therefore, better care options are needed to improve patients’ physical and mental health and unfounded doubts and fears about the effects of chemoradiotherapy.^24^ CBPI is an active, disease-centered treatment strategy that requires good coordination between patients and clinical practitioners.^25^ Psychological intervention and other treatment methods are demonstrated to correct patients’ misunderstandings and facilitate self-management. Clinical nurses implement psychological intervention plans to help patients recognize, assess, and change their maladaptive thoughts related to emotional disturbance^26^ and thereby synchronize their thinking with medical practitioners.

### Limitations

It includes the small size and the short-term follow-up. In the future, more clinical works with a larger sample size and longer follow-up periods are expected to evaluate the treatment strategy with a more robust and objective study design for the best interest of more patients.

## CONCLUSIONS

CBPI is worthy of adoption into clinical practice considering its role in helping patients with NPC in radiotherapy improve social adaptation, psychological resilience, negative emotions like anxiety and depression, confidence in treatment, hope of recovery, physical and mental health, and QoL.

### Authors’ Contributions:

**XL** and **CW:** Carried out the studies, participated in collecting data, drafted the manuscript, are responsible and accountable for the accuracy and integrity of the work; **YL:** Performed the statistical analysis and participated in its design;

**YW:** Participated in acquisition, analysis, or interpretation of data and drafting the manuscript.

All authors read and approved the final manuscript.
